# Microarray Analysis of Circular RNA Expression Profile Associated with 5-Fluorouracil-Based Chemoradiation Resistance in Colorectal Cancer Cells

**DOI:** 10.1155/2017/8421614

**Published:** 2017-06-01

**Authors:** Wei Xiong, Yi-Qin Ai, Yun-Fen Li, Qing Ye, Zheng-Ting Chen, Ji-Yong Qin, Qiu-Yan Liu, Hong Wang, Yun-He Ju, Wen-Hui Li, Yun-Feng Li

**Affiliations:** ^1^Department of Radiation Oncology, Yunnan Cancer Hospital, The Third Affiliated Hospital of Kunming Medical University, Kunming, China; ^2^Department of Radiation Oncology, The First Affiliated Hospital of Kunming Medical University, Kunming, China; ^3^Department of Colorectal Surgery, Yunnan Cancer Hospital, The Third Affiliated Hospital of Kunming Medical University, Kunming, China

## Abstract

Preoperative 5-fluorouracil- (5-FU-) based chemoradiotherapy is a standard treatment for locally advanced colorectal cancer (CRC). However, the effect of 5-FU-based chemoradiotherapy on CRC is limited due to the development of chemoradiation resistance (CRR), and the molecular mechanisms underlying this resistance are yet to be investigated. Recently, circular RNAs (circRNAs), which can function as microRNA sponges, were found to be involved in the development of several cancers. In this study, we focused on clarifying the modulation of the expression profiles of circRNAs in CRR. Microarray analysis identified 71 circRNAs differentially expressed in chemoradiation-resistant CRC cells. Among them, 47 were upregulated and 24 were downregulated by more than twofold. Furthermore, expression modulation of five representative circRNAs was validated by quantitative reverse transcription PCR (qRT-PCR). Moreover, these modulated circRNAs were predicted to interact with 355 miRNAs. Furthermore, Kyoto Encyclopedia of Genes and Genomes (KEGG) pathway analysis showed that the most modulated circRNAs regulate several cancers and cancer-related pathways, and the possible mechanism underlying CRR was discussed. This is the first report revealing the circRNA modulations in 5-FU chemoradiation-resistant CRC cells by microarray. The study provided a useful database for further understanding CRR and presents potential targets to overcome CRR in CRC.

## 1. Introduction

Colorectal cancer (CRC) is one of the most common cancers worldwide, with nearly one million new cases diagnosed every year. CRC is a highly treatable and often curable disease when localized to the bowel [1]. However, even in this situation, there were more than fifty thousand deaths from CRC in the United States in 2015 [2]. Chemoradiotherapy is regarded as a standard treatment for locally advanced CRC, especially, middle and distal rectal cancers [3–5]. For several years, 5-fluorouracil (5-FU) has been the first-choice chemotherapy drug for CRC. However, advances in 5-FU-based chemoradiotherapy of CRC are limited by the development of chemoradiation resistance (CRR) [6]. Hence, clarifying the molecular characteristics is essential to overcome CRR in CRC.

Recently, a novel class of noncoding (nc) RNA, called circular RNA (circRNA), was identified. It is characterized by the presence of a covalent bond linking the 3′ and 5′ ends, which are generated by back splicing [7, 8]. circRNA expression is often cell type-, tissue-, and developmental stage-specific, and several circRNAs are known to be conserved across species [7]. circRNAs can function as protein decoys [9] and as transcriptional regulators, such as EIciRNA, which was shown to enhance transcription of its parent gene [10]. More recently, ciRS-7 was found to act as the inhibitor/sponge of miR-7 [11]. ciRS-7 expression efficiently affects the activity of miR-7, resulting in upregulation of miR-7-targeted transcripts [11, 12]. However, research on circRNAs in CRC [12], especially, CRR in CRC, is limited.

To address the roles of circRNAs in development of CRR in CRC, we performed microarray analysis of 5-FU-resistant cells, CRR-HCT116, along with their parental control cells.

## 2. Materials and Methods

### 2.1. Cell Lines, Cell Culture, and Reagents

The human CRC cell line, HCT116, purchased from China Centre for Type Culture Collection, Chinese Academy of Sciences, was maintained as described previously [6]. The cells were cultured in RPMI medium supplemented with 10% heat-inactivated fetal bovine serum (FBS) and 1% penicillin/streptomycin in a humidified incubator at 37°C with 5% CO_2_ atmosphere (all cell culture reagents were obtained from Thermo Fisher). 5-FU was purchased from Sigma-Aldrich.

### 2.2. Establishment of 5-FU-Based In Vitro CRR Model

5-FU-based in vitro CRR model was established as described previously [6]. HCT116 cells were seeded in 6-well plates at a density of 1 × 10^6^ cells/well and were treated with 10 *μ*mol/L 5-FU and exposed to a single dose of 4 Gy 6 Mv X-ray at room temperature. The cells were incubated for an additional 24 h in the presence of 5-Fu and then incubated in a drug-free culture medium. After 2-3 days, numerous apoptotic cells were observed to be floating in the culture medium. Next, the remaining attached tumor cells were harvested and transferred to the fresh culture medium for recovery and were again subjected to 5-FU and X-ray treatments. Further, this procedure was repeated nine times. Finally, the remaining tumor cells were subcultured to construct the 5-FU-based CRR cell model.

### 2.3. RNA Extraction and RNA Quantity

Total RNA was extracted from snap-frozen HCT116 and CRR-HCT116 cells using TRIzol reagent (Invitrogen, Carlsbad, CA, USA) according to the manufacturer's instructions. The amount and quality of RNA were determined by absorbance ratios, A260/A280 and A260/A230, using NanoDrop ND-1000. RNA integrity was determined by standard agarose gel electrophoresis.

### 2.4. RNA Labeling and Microarray Hybridization

Total RNA from each sample was quantified using the NanoDrop ND-1000. Sample preparation and microarray hybridization were performed according to the standard protocols of Arraystar. Briefly, total RNA was digested with RNase R (Epicentre, Inc.) to remove linear RNAs and enrich circular RNAs. The enriched circular RNAs were then amplified and transcribed into fluorescent cRNA using a random priming method (Arraystar Super RNA Labeling Kit; Arraystar). The RNeasy Mini Kit (Qiagen) was used to purify the labeled cRNAs and the NanoDrop ND-1000 was used to detect the concentration and specific activity of the labeled cRNAs. Five microliter 10x blocking agent and 1 *μ*L 25x fragmentation buffer were added to 1 *μ*L cRNA, and the mixture was incubated at 60°C for 30 min to fragment the labeled cRNA. Besides, 25 *μ*L 2x hybridization buffer was added to dilute the labeled cRNA. Lastly, Arraystar Human circRNA Array (8 × 15K, Arraystar) was used to hybridize the labeled cRNAs. After washing the slides, Agilent Scanner G2505C was used to scan the arrays.

### 2.5. Bioinformatics Analysis

Acquired array images were analyzed using Agilent Feature Extraction software (v 11.0.1.1). Then R software package was used to perform quantile normalization and subsequent data processing. Differentially expressed circRNAs were identified by volcano plot filtering. Distinguishable expression patterns of circRNAs among samples were shown in hierarchical clustering. Besides, differentially expressed circRNAs between two groups were identified using the cut-off of absolute fold change > 2, and *p* < 0.05.

### 2.6. Validation of Differentially Expressed circRNAs by Quantitative Reverse Transcription PCR

Total RNA was extracted from the frozen cells in triplicate using TRIzol reagent (Invitrogen Life Technologies) and then reverse transcribed using a SuperScriptTM III Reverse Transcriptase Kit (Invitrogen) according to the manufacturer's instructions. Quantitative reverse transcription PCR (qRT-PCR) was performed in the ViiATM 7 Real-Time PCR System (Applied Biosystems) instrument using SYBR Green Real-Time PCR Master Mix (TOYOBO, number QPK-201). Each group was studied in triplicate and the primers used for PCR analysis are listed in Table 1.* GAPDH* was used as internal control and fold changes in expression were calculated using ΔΔC_T_ method.

### 2.7. Detecting Putative miRNA Binding Sites

The mature miRNAs only were considered for seed sequence analysis, and FASTA files of miRNAs were obtained from miRBase release 20.0 (http://www.mirbase.org/), and then the miRNAs were aligned with circRNAs. A putative target site of an miRNA is a 6-nucleotide-long sequence in the genome that represents the reverse complement of nucleotides 2–7 of the mature miRNA sequence.

### 2.8. miRNA Target Prediction and Kyoto Encyclopedia of Genes and Genomes Pathway Analysis

The microRNA target prediction and Kyoto Encyclopedia of Genes and Genomes (KEGG) pathway analysis were performed on the website: http://mirsystem.cgm.ntu.edu.tw/index.php [13]. miRsystem is a database that integrates seven well-known miRNA target gene prediction programs: DIANA, miRanda, miRBridge, PicTar, PITA, rna22, and TargetScan. The circRNA-miRNA-gene network was generated using Cytoscape311.

## 3. Results

### 3.1. Expression Profiles of circRNAs

**Figure 1:**
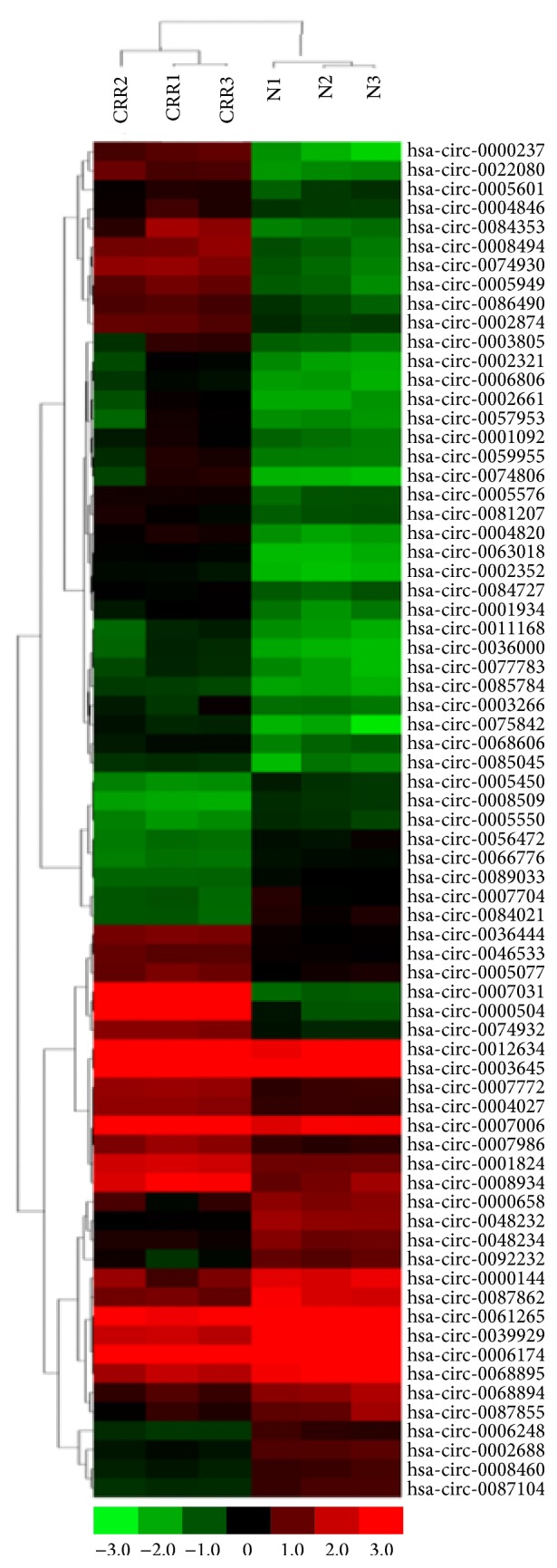
circRNA expression in CRR-HCT116 and parental HCT116 cells. Heat plots of circRNA in CRR-HCT116 and parental HCT116 cells. Each column represents the expression profile of a cell sample, and each row corresponds to a circRNA. “Red” indicates higher expression level, and “green” indicates lower expression level.

After quantile normalization of the raw data, expression profiles of 3731 circRNAs were obtained from CRR-HCT116 and the parental control cells. Differentially expressed circRNAs between the two groups were identified by the cut-off fold change > 2 and *p* < 0.05. Finally, 71 circRNAs were identified, of which 47 were upregulated and 24 were downregulated. A clustered heatmap (Figure 1) showed upregulation or downregulation of circRNAs. The top 10 upregulated and top 5 downregulated circRNAs are shown in Table 2. These data indicated that the majority of modulated circRNAs were upregulated. Additionally, it should be noted that 3 circRNAs were upregulated more than 10-fold, and the top 2 circRNAs were upregulated by 116- and 74-fold, respectively.

**Figure 2:**
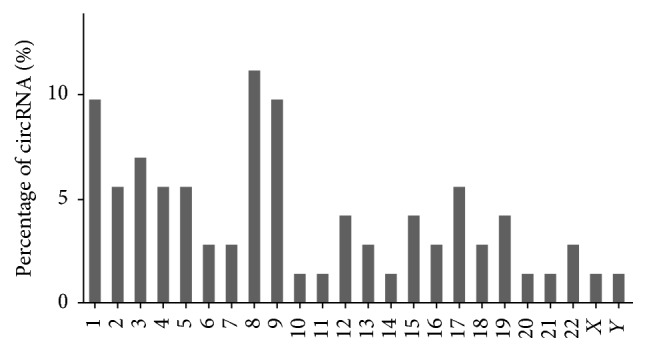
Chromosomal locations of variably expressed circRNA. The *x*-axis represents the ordinal of the chromosome, and the *y*-axis represents the percentage of circRNAs that were expressed differently between CRR-HCT116 and parental HCT116 cells (fold change > 2).

To understand the correlation between the chromosome distribution of CRR and circRNAs, a statistical analysis was performed. According to data in Figure 2, every chromosome has circRNA locations, but chromosomes 1, 8, and 9 have much more circRNA locations than other chromosomes do, and the percentage of circRNAs located on these chromosomes was 11%, 10%, and 10%, respectively. However, only 3% circRNAs, including both of the top two upregulated circRNAs, has_circ_0007031 and has_circ_0000504, were located on chromosome 13 and were spliced from the same parental gene,* TUBGCP3*. This data suggested that chromosomes 1, 8, and 9 have a stronger correlation with CRR than other chromosomes have. However, the top two upregulated circRNAs located on chromosome 13 might be the most important circRNAs in CRR.

### 3.2. Validation of the Microarray Data Using qRT-PCR

**Figure 3:**
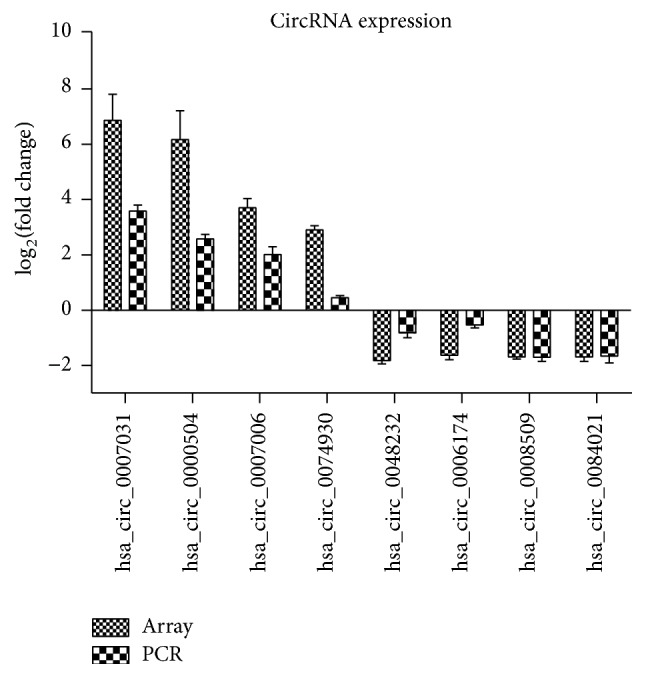
Validation of microarray data by qRT-PCR. Eight differentially expressed circRNAs were validated by qRT-PCR. The heights of the columns in the chart represent the mean expression value of log_2_ fold changes (CRR HCT116/parental HCT116).

To validate the microarray data, four upregulated and four downregulated circRNAs were selected as representatives for further validation by qRT-PCR using primers mentioned in Table 1. According to the data in Figure 3, five of the eight tested circRNAs yielded results quite similar to those of microarray; these well-validated circRNAs included three upregulated circRNAs, hsa_circ_0007031, hsa_circ_0000504, and hsa_circ_0007006. Although the other three circRNAs were not well repeated, the direction of change was similar to that noted in microarray data. This result suggested that most of the circRNAs identified by microarray were reliable and worth being further investigated.

### 3.3. Prediction of miRNAs That Bind to circRNAs

**Figure 4:**
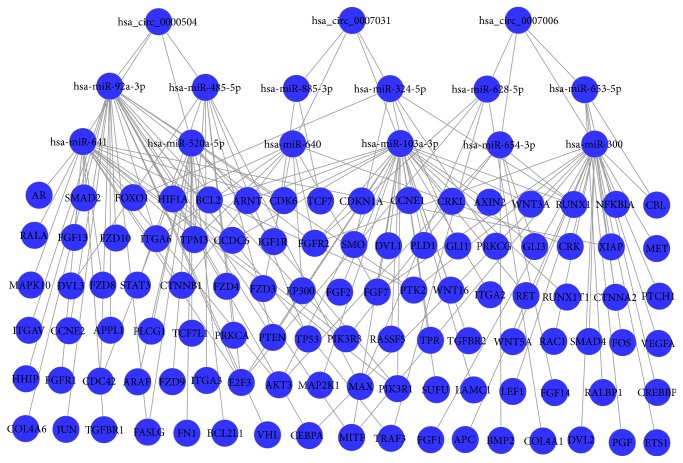
circRNA-miRNA-target gene network of top three upregulated circRNAs in cancer signaling pathways. Interactions between circRNAs and miRNAs and those between miRNAs and target genes in cancer signaling were shown in the map.

Since circRNAs can function as sponges or inhibitors of their interacting miRNAs, circRNAs interacting with miRNAs were predicted. A total of 355 mature miRNAs were predicted to have docking sites in the identified circRNAs, and therefore, they could interact with these circRNAs. The circRNA-miRNA interacting network of the top three upregulated circRNAs was established and shown in Figure 4. It should be noted that different circRNAs can bind to the same miRNAs, which suggested that regulation of miRNAs by circRNAs was complicated. Therefore, it was supposed that different circRNAs can synergistically regulate the activity of specific miRNAs and exert biological roles by indirectly regulating the miRNA target genes.

### 3.4. Prediction of Signaling Pathways and Networks Regulated by circRNAs

**Figure 5:**
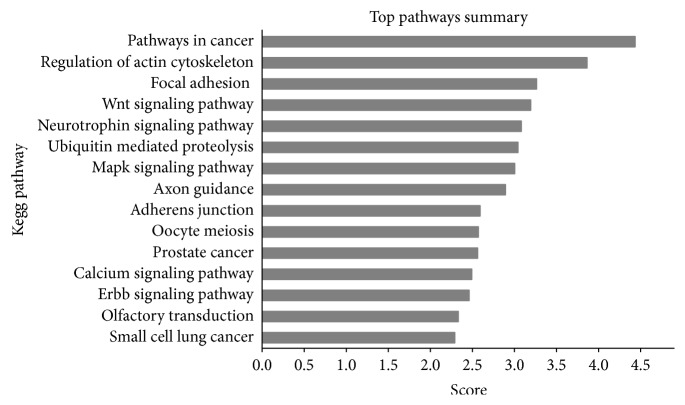
Annotated significant pathways regulated top three upregulated circRNAs. KEGG signaling pathway analysis was performed using miRNAs. The *x*-axis presents score value achieved by miRsystem.

The functional roles of most circRNAs have not yet been defined, but prediction of signaling pathways involving circRNAs by a bioinformatics approach would be beneficial. Therefore, KEGG pathway analysis of the top three upregulated circRNAs was performed by entering information about their interacting miRNAs into miRsystem. The top 15 predicted pathways are shown in Figure 5. Among these pathways, some are directly linked to cancer pathogenesis, such as the prostate cancer pathway and small cell lung cancer signaling. Interestingly, although other pathways, such as actin-cytoskeleton pathway [14] and focal adhesion signaling [15, 16], seem to be not directly linked to CRC, they were also found associated with cancer development.

Since the cancer signaling pathway was the most significant in the predicted results, miRNAs that are directly involved in cancer signaling were analyzed, and brief results are shown in Table S1 (see Supplementary Material available online at https://doi.org/10.1155/2017/8421614). Results suggested that some miRNAs, such as hsa-miR-9a-3p, hsa-miR-103a-3p, and hsa-miR-300, might play more important roles than others since they have more target genes in the cancer pathways (detailed data not shown).

Taken together, in this study, the above results indicated that newly identified circRNAs in CRR were involved in CRC development.

## 4. Discussion

5-FU-based concurrent chemoradiation is recommended as the standard treatment for CRC, but CRR development limited the effect of this treatment. Moreover, the molecular mechanisms underlying 5-FU-based CRR in CRC cells are yet unclear. Thus, understanding the mechanism underlying CRR development is very crucial and essential to overcome the problem. circRNAs were recently identified as novel functional ncRNAs involved in several cancers [7], including CRC. hsa_circ_001569 was reported to act as a positive regulator of proliferation and invasion of CRC cells; it functions as a sponge of miR-145, and therefore, targets upregulated miR-145, such as E2F5, BAG4, and FMNL2 [17]. However, large-scale identification of circRNA expression in chemoradiation-resistant CRC cells was not yet reported.

Previously, we established 5-FU-based chemoradiation-resistant CRC model and identified novel long ncRNAs and mRNAs associated with CRR. These genes were found to be involved in the Jak-STAT, PI3 K-Akt, and NF-*κ*B signaling pathways [6]. These findings provided an advantage to further study the role of circRNAs in CRR development. Here, we first reported microarray analysis of circRNA expression modulations in chemoradiation-resistant CRC cells.

In this study, we found that 47 circRNAs were upregulated and 24 circRNAs were downregulated by more than twofold. The top three upregulated circRNAs were upregulated by more than 10-fold, and the most upregulated circRNA, hsa_circ_0007031, was upregulated by 116-fold, which was a huge modulation. Furthermore, selected representative modulation in circRNAs was validated to be consistent with microarray data by qRT-PCR analysis, suggesting that the array data was reliable. KEGG pathway analysis revealed that modulated circRNAs in 5-FU chemoradiation-resistant CRC cells were involved in a cancer signaling pathway or cancer-related signaling pathways, such as the actin-cytoskeleton pathway [14], focal adhesion signaling [15, 16], and WNT signaling pathway, all of which are associated with CRC development. WNT signaling was known to be associated with CRC for a long time; Wnt2 was expressed at low levels in the normal colon. It was overexpressed in all the tumor tissue samples at the different Dukes' stages of CRC progression [18]. Since the above circRNAs were identified from 5-FU chemoradiation-resistant CRC cells, we supposed that these pathways play important roles in the development of CRR.

The expression profiles of miR-885-3p could significantly (*p* = 0.012) distinguish plasma samples collected prior to treatment from those collected after two days of chemoradiotherapy, suggesting that miR-885-3p is involved in the development of CRR [19]. Because miR-885-3p targets hsa_circ_0007031, which was found highly upregulated in this study, we inferred that hsa_circ_0007031 might play a crucial role in the development of CRR. Meanwhile, it was known that STAT3 [20] plays an important role in the development of CRR, and silencing STAT3 resulted in significantly decreased clonogenic survival following exposure to 5-FU and irradiation. Interestingly, STAT3 was a target gene of hsa-miR-485-5p, which could interact with hsa_circ_0000504, and the latter one was found to be highly upregulated in this study. Thus, we supposed that upregulation of hsa_circ_0000504 could reduce the suppression of hsa-miR-485-5p on STAT3 and accelerate the development of CRR. We speculated that downregulation of hsa_circ_0000504 would be a possible option to overcome 5-FU resistance in CRC.

AKT signaling was reported to be associated with chemoradiotherapy treatment response [21]. In this study, all the top 3 upregulated circRNAs were predicted to be capable of regulating AKT3 by interacting with AKT3 regulatory miRNAs; thus, AKT signaling may be stabilized by these circRNAs. Furthermore, BCL2 protein family [22] was reported to be associated with rectal tumors in patients unresponsive towards chemoradiotherapy. Coincidentally, BCL2 was also predicted to be regulated by both of the top two upregulated circRNAs—hsa_circ_0007031 and hsa_circ_0000504.

Among the downregulated circRNAs, it was found that hsa_circ_0048234 has four miR-671-5p-binding sites and was modulated in chemoradiation-exposed rectal cancer cells. The miR-671-5p-EGFR signaling pathway was identified previously [23]; thus, it was possible that downregulation of hsa_circ_0048234 could, therefore, increase EGFR signaling and promote CRR.

In summary, we first reported the role of differentially expressed circRNAs in 5-FU chemoradiation-resistant CRC cell line. The study provided a useful database for further understanding CRR and presents potential targets to overcome CRR in CRC.

## Supplementary Material

Table S1: Involvement of interacting miRNAs of the top three upregulated circRNAs in cancer pathway.

## Figures and Tables

**Table 1:** The list of primers used in the study.

Name	Primer sequence
GAPDH(HUMAN)	F: 5′-GGGAAACTGTGGCGTGAT-3′
	R: 5′-GAGTGGGTGTCGCTGTTGA-3′
hsa_circ_0007031	F: 5′-ACATCACATTTGAGGTGCTTG-3′
	R: 5′-AAAAGGACCTTCCTAGACTGATC-3′
hsa_circ_0007006	F: 5′-TGTCGGCACAGTTTCGTTCTC-3′
	R: 5′-TTGATCTGGAAGGCATGTGGA-3′
hsa_circ_0074930	F: 5′-GGAAAGGGCTTGATGGAGATT-3′
	R: 5′-TCGCAGTACAGGTGGTTGGA-3′
hsa_circ_0000504	F: 5′-GCAAAGCTCCTGAGAAACAT-3′
	R: 5′-AAAAGGACCTTCCTAGACTGAT-3′
hsa_circ_0048232	F: 5′-TCGGAGTGGTCACGGAGGTA-3′
	R: 5′-CGAGACTGGTTGGTGGTTTTATC-3′
hsa_circ_0006174	F: 5′-CATCCATCACTCCAGCATCAG-3′
	R: 5′-GGTCACCATAACCACCACAAAG-3′
hsa_circ_0008509	F: 5′-CACCATTCATTTACAGGGCACA-3′
	R: 5′-CGCTTGTGGCCTGATTTTG-3′
hsa_circ_0084021	F: 5′-CAGCAAGATCACCGTGAGCATA-3′
	R: 5′-CAGGGCATTGATAACAAAGCAA-3′

**Table 2:** Top modulated circRNAs in chemoradiation-resistant colorectal cancer.

circRNA	Gene symbol	Chrom	Regulation	*p* value	FC (abs)
hsa_circ_0007031	TUBGCP3	chr13	Up	2.29*E* − 04	116.6
hsa_circ_0000504	TUBGCP3	chr13	Up	6.96*E* − 04	74.4
hsa_circ_0007006	DYM	chr18	Up	7.58*E* − 05	13
hsa_circ_0000237	HNRNPF	chr10	Up	2.26*E* − 04	8.7
hsa_circ_0074930	SLIT3	chr5	Up	8.33*E* − 05	7.6
hsa_circ_0084353	PRKDC	chr8	Up	3.61*E* − 03	6.8
hsa_circ_0022080	NUP160	chr11	Up	6.79*E* − 05	6.2
hsa_circ_0008494	ARID1A	chr1	Up	1.82*E* − 04	6.2
hsa_circ_0005949	ZNF608	chr5	Up	2.73*E* − 04	5.6
hsa_circ_0074806	CLINT1	chr5	Up	5.74*E* − 03	4.5
hsa_circ_0048232	DAZAP1	chr19	Down	1.76*E* − 05	3.3
hsa_circ_0006174	RAD23B	chr9	Down	5.28*E* − 04	2.7
hsa_circ_0008509	NAV3	chr12	Down	2.84*E* − 05	2.6
hsa_circ_0084021	PLEKHA2	chr8	Down	3.80*E* − 04	2.6
hsa_circ_0087862	RAD23B	chr9	Down	1.49*E* − 03	2.6

## References

[1] Haggar F. A., Boushey R. P. (2009). Colorectal cancer epidemiology: incidence, mortality, survival, and risk factors. *Clinics in Colon and Rectal Surgery*.

[2] Siegel R. L., Miller K. D., Jemal A. (2015). Cancer statistics, 2015. *CA: Cancer Journal for Clinicians*.

[3] Wan J., Gai Y., Li G., Tao Z., Zhang Z. (2015). Incidence of chemotherapy- and chemoradiotherapy-induced amenorrhea in premenopausal women with stage II/III colorectal cancer. *Clinical Colorectal Cancer*.

[4] Appelt A. L., Pløen J., Harling H. (2015). High-dose chemoradiotherapy and watchful waiting for distal rectal cancer: A prospective observational study. *The Lancet Oncology*.

[5] Lee J. H., Chie E. K., Kim K. (2013). The influence of the treatment response on the impact of resection margin status after preoperative chemoradiotherapy in locally advanced rectal cancer. *BMC Cancer*.

[6] Xiong W., Jiang Y.-X., Ai Y.-Q. (2015). Microarray analysis of long non-coding RNA expression profile associated with 5-fluorouracil-based chemoradiation resistance in colorectal cancer cells. *Asian Pacific Journal of Cancer Prevention*.

[7] Ebbesen K. K., Kjems J., Hansen T. B. (2016). Circular RNAs: identification, biogenesis and function. *Biochimica et Biophysica Acta*.

[8] Chen I., Chen C.-Y., Chuang T.-J. (2015). Biogenesis, identification, and function of exonic circular RNAs. *Wiley Interdisciplinary Reviews: RNA*.

[9] Ashwal-Fluss R., Meyer M., Pamudurti N. R. (2014). CircRNA biogenesis competes with pre-mRNA splicing. *Molecular Cell*.

[10] Li Z., Huang C., Bao C. (2015). Exon-intron circular RNAs regulate transcription in the nucleus. *Nature Structural and Molecular Biology*.

[11] Hansen T. B., Jensen T. I., Clausen B. H. (2013). Natural RNA circles function as efficient microRNA sponges. *Nature*.

[12] Peng L., Yuan X. Q., Li G. C. (2015). The emerging landscape of circular RNA ciRS-7 in cancer (Review). *Oncology Reports*.

[13] Shin E.-Y., Lee B.-H., Yang J.-H. (2000). Up-regulation and co-expression of fibroblast growth factor receptors in human gastric cancer. *Journal of Cancer Research and Clinical Oncology*.

[14] Park Y., Kang M. H., Seo H. Y. (2010). Bone morphogenetic protein-2 levels are elevated in the patients with gastric cancer and correlate with disease progression. *Medical Oncology*.

[15] Zhang X. T., Ni Z. H., Duan Z. P. (2015). Overexpression of E2F mRNAs associated with gastric cancer progression identified by the transcription factor and miRNA co-regulatory network analysis. *PLoS ONE*.

[16] Yang T., Thakur A., Chen T. (2015). MicroRNA-15a induces cell apoptosis and inhibits metastasis by targeting BCL2L2 in non-small cell lung cancer. *Tumor Biology*.

[17] Xie H., Ren X., Xin S. (2016). Emerging roles of circRNA_001569 targeting miR-145 in the proliferation and invasion of colorectal cancer. *Oncotarget*.

[18] Vider B.-Z., Zimber A., Chastre E. (1996). Evidence for the involvement of the Wnt 2 gene in human colorectal cancer. *Oncogene*.

[19] Summerer I., Niyazi M., Unger K. (2013). Changes in circulating microRNAs after radiochemotherapy in head and neck cancer patients. *Radiation Oncology*.

[20] Spitzner M., Roesler B., Bielfeld C. (2014). STAT3 inhibition sensitizes colorectal cancer to chemoradiotherapy in vitro and in vivo. *International Journal of Cancer*.

[21] Hildebrandt M. A. T., Yang H., Hung M.-C. (2009). Genetic variations in the PI3K/PTEN/AKT/mTOR pathway are associated with clinical outcomes in esophageal cancer patients treated with chemoradiotherapy. *Journal of Clinical Oncology*.

[22] Flanagan L., Lindner A. U., de Chaumont C. (2015). BCL2 protein signalling determines acute responses to neoadjuvant chemoradiotherapy in rectal cancer. *Journal of Molecular Medicine*.

[23] Ragusa M., Majorana A., Statello L. (2010). Specific alterations of microRNA transcriptome and global network structure in colorectal carcinoma after cetuximab treatment. *Molecular Cancer Therapeutics*.

