# Supplementary material for: Microarray Analysis of Circular RNA Expression Profile Associated with 5-Fluorouracil-Based Chemoradiation Resistance in Colorectal Cancer Cells
Source: Biomed Res Int. 2017 Jun 1;2017:8421614. doi: 10.1155/2017/8421614 (PMC5471554; doi:10.1155/2017/8421614)
Supplement: Supplementary file 1 — Table S1: Involvement of interacting miRNAs of the top three upregulated circRNAs in cancer pathway. [file 8421614.f1.docx]

Table S1. Involvement of interacting miRNAs of the top three upregulated circRNAs in cancer pathway.

| CircRNAs | MicroRNAs | Pathway in cancer |
| --- | --- | --- |
| hsa_circ_0007031 | hsa_miR_103a_3p | √ |
|  | hsa_miR_194_3p |  |
|  | hsa_miR_324_5p | √ |
|  | hsa_miR-640 | √ |
|  | hsa_miR-885_3p | √ |
| hsa_circ_0000504 | hsa_miR_485_5p | √ |
|  | hsa_miR_520a_5p | √ |
|  | hsa_miR_641 | √ |
|  | hsa_miR_659_5p |  |
|  | hsa_miR_92a_3p | √ |
| hsa_circ_0007006 | hsa_miR_16_2_3p |  |
|  | hsa_miR_300 | √ |
|  | hsa_miR_628_5p | √ |
|  | hsa_miR_653_5p | √ |
|  | hsa_miR_654_3p | √ |
